# Development and internal validation of a model predicting severe maternal morbidity using pre-conception and early pregnancy variables: a population-based study in Ontario, Canada

**DOI:** 10.1186/s12884-021-04132-6

**Published:** 2021-10-06

**Authors:** Natalie Dayan, Gabriel D. Shapiro, Jin Luo, Jun Guan, Deshayne B. Fell, Carl A. Laskin, Olga Basso, Alison L. Park, Joel G. Ray

**Affiliations:** 1 Department of Medicine and Research Institute, McGill University Health Centre, 5252 de Maisonneuve West, 2B.40, Montreal, QC H4A 3S5 Canada; 2 Department of Epidemiology, Biostatistics and Occupational Health, McGill University, Purvis Hall, 1020 Pine Ave West, Montreal, QC H3A 1A2 Canada; 3 ICES, 2075 Bayview Ave, Toronto, ON M4N 3M5 Canada; 4 School of Epidemiology and Public Health, Faculty of Medicine, University of Ottawa, Centre for Practice-Changing Research Building, Room L-1154, 401 Smyth Road, Ottawa, ON K1H 8L1 Canada; 5 Departments of Medicine and Obstetrics and Gynecology, University of Toronto, 123 Edward St., suite 1200, Toronto, ON M5G 1E2 Canada; 6 TRIO Fertility, 655 Bay St, Toronto, ON M5G 2K4 Canada

**Keywords:** Women’s health, Epidemiology, Obstetrics, Gynaecology

## Abstract

**Background:**

Improvement in the prediction and prevention of severe maternal morbidity (SMM) - a range of life-threatening conditions during pregnancy, at delivery or within 42 days postpartum - is a public health priority. Reduction of SMM at a population level would be facilitated by early identification and prediction. We sought to develop and internally validate a model to predict maternal end-organ injury or death using variables routinely collected during pre-pregnancy and the early pregnancy period.

**Methods:**

We performed a population-based cohort study using linked administrative health data in Ontario, Canada, from April 1, 2006 to March 31, 2014. We included women aged 18–60 years with a livebirth or stillbirth, of which one birth was randomly selected per woman. We constructed a clinical prediction model for the primary composite outcome of any maternal end-organ injury or death, arising between 20 weeks’ gestation and 42 days after the birth hospital discharge date. Our model included variables collected from 12 months before estimated conception until 19 weeks’ gestation. We developed a separate model for parous women to allow for the inclusion of factors from previous pregnancy(ies).

**Results:**

Of 634,290 women, 1969 experienced the primary composite outcome (3.1 per 1000). Predictive factors in the main model included maternal world region of origin, chronic medical conditions, parity, and obstetrical/perinatal issues – with moderate model discrimination (C-statistic 0.68, 95% CI 0.66–0.69). Among 333,435 parous women, the C-statistic was 0.71 (0.69–0.73) in the model using variables from the current (index) pregnancy as well as pre-pregnancy predictors and variables from any previous pregnancy.

**Conclusions:**

A combination of factors ascertained early in pregnancy through a basic medical history help to identify women at risk for severe morbidity, who may benefit from targeted preventive and surveillance strategies including appropriate specialty-based antenatal care pathways. Further refinement and external validation of this model are warranted and can support evidence-based improvements in clinical practice.

**Supplementary Information:**

The online version contains supplementary material available at 10.1186/s12884-021-04132-6.

## Background

Severe maternal morbidity (SMM) covers a range of conditions along the continuum to maternal death during pregnancy or within 42 days after delivery [1]. Maternal morbidity is a substantial public health concern [2] whose incidence is rising in Canada and the US [3]. These patterns are driven by multiple risk factors including delaying childbearing, use of assisted reproductive technologies, rising rates of obesity, and Caesarean delivery [4]. Because of the low prevalence of maternal mortality in many industrialized countries, data covering several years are required to compute precise estimates of prevalence and risk factors, thus complicating the use of maternal mortality as a population health indicator [2, 5]. Consequently, SMM has received increasing attention as an indicator of perinatal health and obstetric care [6, 7].

As the focus in industrialized countries such as Canada has shifted towards ‘near miss’ events as a means to improving the health and quality of care for pregnant women [5], prediction of SMM has been identified as a critical research gap in obstetrics [4]. Many maternal characteristics are known pre-conception or early in pregnancy and are strong risk factors for the development of SMM [2, 8]. Therefore, a combination of such factors may reliably predict its onset, enabling evidence-based and rational early triage of high-risk women for enhanced surveillance and subspecialty-based care.

Advances in maternal morbidity risk prediction include a US obstetric comorbidity index [9], which was externally validated within a Canadian population, resulting in modest discrimination (C-statistic of 0.66, 95% confidence interval [CI] 0.65–0.67) [10]. That index included variables that both preceded, and were simultaneous with, the onset of SMM, making it a useful research tool for identifying the burden of morbidity but less so for clinical prediction. Others have developed models focused on specific subtypes of maternal morbidity, such as cardiovascular-related conditions [7]. Models predicting maternal mortality include the Collaborative Integrated Pregnancy High-dependency Estimate of Risk (CIPHER) model (C-statistic 0.82, 95% CI 0.81–0.84) and the Maternal Severity Index (C-statistic 0.83, 95% CI 0.80–0.85) [11], both developed among women either already critically ill or hospitalized, and mostly later in gestation.

Since SMM predominantly arises around birth or early postpartum [1], the ideal timeframe for prediction is before or early in pregnancy to facilitate effective preventive strategies such as referral to high-risk centres or shared-care antenatal care pathways [12, 13]. Existing models do not enable these latter steps, nor do they account for important pre-pregnancy factors, such as maternal infertility and its treatment, which are associated with SMM [14]. Additionally, existing prediction efforts did not consider prior adverse pregnancy outcomes among parous women. We therefore undertook the current study to develop and internally validate a clinical prediction model of SMM, defined as a composite of maternal end-organ injury or death, using readily available factors ascertained pre-pregnancy and prior to 20 weeks’ gestation in a population-based study in Ontario – Canada’s most populous and multi-ethnic province.

## Methods

The use of data in this project was authorized under section 45 of Ontario’s Personal Health Information Protection Act, which does not require review by a Research Ethics Board. We followed the Transparent reporting of a multivariable prediction model for individual prognosis or diagnosis (TRIPOD) guideline for reporting of prediction studies [15].

### Population and data sources

All women with a pregnancy lasting beyond 20 weeks’ gestation, and who delivered within an Ontario hospital between April 1, 2006 and March 31, 2014, were identified within the Better Outcomes Registry & Network (BORN) databases [16]. Data beyond 2014 were not available in these datasets. The BORN registry captures over 99% of hospital births in the province, and has been validated for data completeness and accuracy [17, 18]. We used the Registered Persons Database, the Immigration, Refugees and Citizenship Canada’s Permanent Resident Database, the Ontario Health Insurance Plan (OHIP) outpatient claims database, and the Canadian Institute for Health Information (CIHI) Discharge Abstract Database to capture maternal demographics, pre-existing health conditions and diagnoses and procedures documented during a hospitalization (see Table S1 for variables and diagnostic codes used to develop the study cohort). The datasets were linked using unique encoded identifiers and analysed at ICES – a not-for-profit provincial research entity that houses a large network of health administrative databases (https://www.ices.on.ca/).

We excluded ectopic pregnancies, pregnancies resulting in abortion or miscarriage or ending before 20 weeks’ gestation. We randomly sampled one birth (live- or stillbirth) per woman to avoid potential within-person correlations among women with multiple pregnancies (Table S1; Figure S1).

### Study outcomes

The primary composite outcome was maternal end-organ injury or death arising between 20 weeks’ gestation and up to 42 days after the index birth hospital discharge date. The list of conditions used to define maternal end-organ injury was based on the model developed by Bateman [9] and validated by Metcalfe [10], comprising 20 diagnoses and procedures, and consistent with Canadian perinatal surveillance definitions for SMM and death [19–21] (Table S1).

A secondary outcome was all-cause maternal mortality, from birth until 365 days postpartum, since previous work has shown a persistent increase in mortality risk beyond the early postpartum period [22, 23].

### Candidate predictors, variable selection, and coding

Demographic, medical and obstetric factors known to be associated with an increased risk of SMM were considered as candidate predictors. These included: estimated maternal age at conception (continuous, categorical, and squared terms); residential income quintile; world region of origin (Table S2), as a proxy for both maternal birthplace and ethnicity; attendance at a first-trimester prenatal care visit; pre-pregnancy body mass index (BMI); parity; multiple gestation; infertility; infertility treatment; placental disorders (e.g., placenta praevia, placenta accreta); and pre-existing medical conditions coded within 12 months before the estimated date of conception (Table S1). Substantial missing data were noted only for the variable pre-pregnancy BMI (63.8%). We tested models in which BMI was modelled as a continuous variable and where missing values were assigned the median BMI (24.2 kg/m^2^). We further tested models in which BMI was divided into the following categories: < 18.5 kg/m^2^, 18.5–24.9 kg/m^2^ (reference category), 25–29.9 kg/m^2^, > 30 kg/m^2^, and missing. Certain categorical variables with a low frequency in the cohort were combined with other similar variables (e.g., pre-existing cardiovascular conditions; placental conditions and anomalies). Variables were also assessed for collinearity by checking the variance inflation factor (VIF), and where collinear (VIF > 5), the most commonly reported variable was selected [24].

In the model restricted to the sub-cohort of parous women, in addition to the above variables, we included complications coded in any previous pregnancy as predictors (Table S1).

Possible interactions between variables were assessed and included if statistically significant at alpha = 0.10 [25].

### Statistical analysis

#### Descriptive statistics

We used standardized differences to contrast births with and without the primary composite outcome of maternal end-organ injury or death, with a value > 0.10 indicating an important difference in baseline characteristics [26].

#### Model discrimination

Among the entire cohort, a logistic regression model was fit using the final selected variables to predict the primary composite outcome of maternal acute end-organ injury or death from 20 weeks’ gestation until 42 days postpartum. A backward elimination method was applied for variable selection, with predictor evaluation based on a balance of the model’s C-statistic, clinical influence, and statistical significance. For continuous predictor variables such as age in which non-linear associations with the outcome were observed, a quadratic (squared) term was added to the model. Model discrimination was expressed as a C-statistic and its 95% CI, as well as visual detection of a receiver operating curve (ROC). We considered a C-statistic of < 0.5 to be not useful, 0.5 to 0.6 poor, 0.6 to 0.7 moderate, and ≥ 0.7 as good [27].

#### Model internal validation

To arrive at an optimism-corrected C-statistic, we used a bootstrapping approach, with 500 bootstrap samples selected from the original cohort, with replacement [28] – an approach known to produce stable estimates with low risk of bias [29]. The optimism-corrected C-statistic was defined as the C-statistic from the original data minus the optimism value [30].

#### Model calibration

Model calibration was assessed by visual inspection of calibration plots of observed vs. expected probabilities of the outcome, where a 45-degree line denotes good calibration, and a slope of 1 indicates perfect agreement between observed and expected events [31].

#### Risk classification

We used a risk classification table and computed likelihood ratios (LRs) [32] with associated 95% CI to assess the main model’s ability to stratify the population as low or high-risk. We divided the population into five groups of predicted probability: very low risk (< 1.5 per 1000), low risk (1.5 to 3 per 1000), intermediate risk (3 to 5 per 1000), high risk (5 to 15 per 1000) and very high risk (> 15 per 1000). These cut-offs were chosen based on the overall incidence of our primary outcome of 3.1 per 1000, which we assumed to reflect the risk among the majority of the cohort. Positive LRs of > 5 and > 10 were interpreted as moderately or very useful “rule-in” tests, while values between 0.2 and 0.5, and < 0.1 were considered moderately and very useful “rule-out” tests [33].

### Funding

This study was supported by funding from the Canadian Institutes of Health Research (grant number 15139).

## Results

After sampling one birth per woman from among 853,517 eligible births, the total cohort comprised 634,290 births (Figure S1). The primary outcome of end-organ injury or death from 20 weeks’ gestation up to 42 days postpartum occurred in 1969 women (3.1 per 1000), including 62 deaths (0.1 per 1000). Women who experienced the primary outcome were older, more likely to have a pre-existing medical condition, and to have had infertility treatment (Table 1).

**Table 1:** Baseline characteristics of the study population, according to whether a woman had the composite outcome of maternal end-organ injury or death between 20 weeks’ gestation and up to 42 days after birth. All data are shown as a number (%) unless otherwise stated

**Characteristic**	**With maternal end-organ injury or death**^**a**^** (*N* = 1969)**	**Without maternal end-organ injury or death (*N* = 632,321)**	**Standardized difference**
**Demographic factors, at conception**			
Age, y			
Mean ± SD	31.0 ± 5.9	29.6 ± 5.5	0.24
18–34	1414 (71.8)	512,340 (81.0)	0.22
35–39	428 (21.7)	100,490 (15.9)	0.15
40–44	112 (5.7)	18,529 (2.9)	0.14
≥ 45	15 (0.8)	962 (0.2)	0.09
Neighbourhood income quintile (Q)			
Q1 (lowest)	521 (26.5)	145,947 (23.1)	0.08
Q2	388 (19.7)	128,604 (20.3)	0.02
Q3	422 (21.4)	129,051 (20.4)	0.03
Q4	370 (18.8)	128,928 (20.4)	0.04
Q5 (highest)	268 (13.6)	99,791 (15.8)	0.06
World region of origin			
Western Nations and Europe	69 (3.5)	27,845 (4.4)	0.05
Hispanic America	47 (2.4)	14,183 (2.2)	0.01
Caribbean	60 (3.0)	10,305 (1.6)	0.09
Sub-Saharan Africa	80 (4.1)	12,073 (1.9)	0.13
Middle East and North Africa	43 (2.2)	18,138 (2.9)	0.04
East Asia and Pacific	139 (7.1)	42,556 (6.7)	0.01
South Asia	166 (8.4)	55,899 (8.8)	0.01
Canada/Long-term resident	1365 (69.3)	451,322 (71.4)	0.04
**Infertility history identified in the index pregnancy**^**b**^	254 (12.9)	49,231 (7.8)	0.17
Any infertility treatment	110 (5.6)	18,449 (2.9)	0.13
IVF^c^	80 (72.7)	9830 (53.3)	0.41
Non-IVF^c^	30 (27.3)	8619 (46.7)	0.41
**Obstetrical factors in the index pregnancy**			
Parity			
0	1004 (51.0)	299,851 (47.4)	0.07
1–2	823 (41.8)	297,303 (47.0)	0.11
≥ 3	142 (7.2)	35,167 (5.6)	0.07
Multiple gestation	179 (9.1)	18,149 (2.9)	0.26
Had a first-trimester prenatal visit	1296 (65.8)	457,620 (72.4)	0.14
Placental disorder^d^	160 (8.1)	12,020 (1.9)	0.29
Median (IQR) gestational age at birth, weeks	38 (36–39)	39 (38–40)	0.51
**Obstetrical factors identified in any prior pregnancy**^**e**^			
Unplanned Caesarean birth	340 (35.2)	65,205 (19.6)	0.36
Severe organ injury	21 (1.1)	482 (0.1)	0.13
Preeclampsia, eclampsia or HELLP syndrome	29 (3.0)	5038 (1.5)	0.1
Preterm birth < 37 weeks’ gestation	90 (9.3)	19,958 (6.0)	0.13
Gestational diabetes mellitus	57 (5.9)	12,512 (3.8)	0.1
Previous spontaneous abortion			
0	1426 (72.4)	487,379 (77.1)	0.11
1–2	486 (24.7)	131,660 (20.8)	0.09
≥ 3	57 (2.9)	13,167 (2.1)	0.05
Missing	≤5 (0.0)	116 (0.0)	0.02
**Medical factors identified within 365 days before the estimated date of conception in the index birth**			
Median (IQR) body mass index, kg/m^2f^	24.2 (24.2–24.2)	24.2 (24.2–24.2)	0.10
Body mass index category			
< 18.5 kg/m^2^	34 (1.7)	11,622 (1.8)	0.01
18.5–24.9 kg/m^2^	356 (18.1)	116,614 (18.4)	0.01
25–29.9 kg/m^2^	212 (10.8)	56,016 (8.9)	0.06
> 30 kg/m^2^	202 (10.3)	44,644 (7.1)	0.11
Missing	1165 (59.2)	403,425 (63.8)	0.1
Obese (body mass index > 30 kg/m^2^ at any visit)^f^	239 (12.1)	54,493 (8.6)	0.12
Median (IQR) body mass index, kg/m^2^ (without replacement)^g^	25.2 (22.0–30.1)	24.2 (21.3–28.5)	0.17
Chronic hypertension	156 (7.9)	16,796 (2.7)	0.24
Renal disease	27 (1.4)	758 (0.1)	0.15
Diabetes mellitus (non-gestational)	94 (4.8)	10,203 (1.6)	0.18
Dyslipidemia	51 (2.6)	12,161 (1.9)	0.04
Cardiovascular morbidity^h^	122 (6.2)	12,469 (2.0)	0.21
Systemic lupus erythematosus	14 (0.7)	722 (0.1)	0.09
Asthma	213 (10.8)	43,376 (6.9)	0.14
Other medical conditions^i^	7 (0.4)	709 (0.1)	0.05
Alcohol overuse	14 (0.7)	2654 (0.4)	0.04
Any substance use	76 (3.9)	12,295 (1.9)	0.11
Any tobacco use	231 (11.7)	72,387 (11.4)	0.01

*Abbreviations*: *SD* Standard deviation, *IQR* Interquartile range, *IVF* In vitro fertilization, *HELLP* Haemolysis elevated liver enzymes low platelets

^a^Maternal end-organ injury or death occurring from 20 weeks’ gestation to 42 days after birth

^b^Includes diagnosis of infertility, endometriosis, and polycystic ovarian syndrome

^c^Percentages are among those who received any infertility treatment

^d^Includes placenta praevia, placenta accreta, vasa praevia, and other placental disorders

^e^Among 333,435 parous women

^f^Body mass index was known among 804 (40.8%) women with the composite outcome, and 228,896 (36.2%) who did not have the composite outcome. For women with a missing body mass index, or an implausible value < 10 or > 50 kg/m^2^, a median body mass index of 24.2 kg/m^2^ was used

^g^Women with a missing body mass index, or an implausible value < 10 or > 50 kg/m^2^, were removed

^h^Includes chronic congestive heart failure, congenital heart disease, pulmonary hypertension, coronary artery disease, cardiac dysrhythmia, chronic rheumatic heart diseases, or non-incident stroke or myocardial infarction within the previous 365 days

^i^Includes sickle cell disease and HIV

The most frequent factors contributing to end-organ injury or death were acute heart failure (40.6%), need for assisted ventilation (29.2%), acute renal failure (12.0%) and shock (10.1%) (Table 2).

**Table 2:** Occurrence of maternal end-organ injury or death between 20 weeks’ gestation and up to 42 days after birth, and the ranking of the most prevalent morbidity indicators

**Outcome**	**Number of outcome events**	**Rate per 1000**	**Proportion of all outcomes (%)**^**a**^
Maternal end-organ injury or death between 20 weeks’ gestation and 42 days after birth	1969	3.1	100.0
Maternal end-organ injury, without death, between 20 weeks’ gestation and 42 days after birth	1907	3.0	96.9
Death between 20 weeks’ gestation and 42 days after birth	62	0.1	3.1
Death without end-organ injury	19	0.03	1.0
Combined maternal end-organ injury *and* death between 20 weeks’ gestation and 42 days after birth	43	0.07	2.2
Acute heart failure	800	1.3	40.6
Assisted ventilation through endotracheal tube	575	0.9	29.2
Acute renal failure	237	0.4	12.0
Shock	198	0.3	10.1
Adult respiratory distress syndrome or respiratory failure	157	0.3	8.0
Puerperal cerebrovascular disorders	134	0.2	6.8
Acute liver disease	85	0.1	4.3
Disseminated intravascular coagulation	54	0.09	2.7
Acute psychosis/delirium	49	0.08	2.5
Dialysis	37	0.06	1.9
Sepsis	33	0.05	1.7
Acute myocardial infarction	32	0.05	1.6
Left ventricular failure	32	0.05	1.6
Status epilepticus	23	0.04	1.2
Status asthmaticus	22	0.03	1.1
Cerebral oedema or coma	20	0.03	1.0
Assisted ventilation through tracheostomy	8	0.01	0.4

^a^Categories not mutually exclusive

### Model discrimination and internal validation

#### Overall cohort

**Fig. 1:**
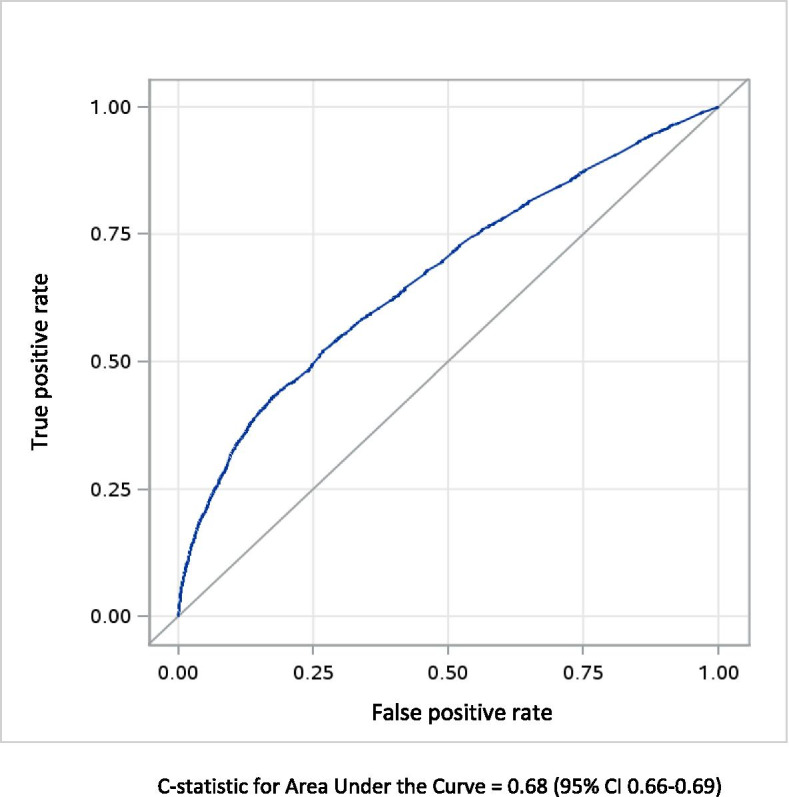
Receiver operating characteristic curve showing discrimination of the clinical prediction model for maternal end-organ injury or death. Legend: Outcomes are those arising between 20 weeks’ gestation and 42 days after birth, using variables measured pre-pregnancy, and in the index pregnancy prior to 20 weeks’ gestation. Predictor variables and adjusted odds ratios are shown in Table S3. Analysed is the entire cohort of 634,290 births. C-statistic for Area Under the Curve = 0.68 (95% CI 0.66–0.69)

In the overall cohort (*n* = 634,290), variables significantly associated with the composite outcome of maternal end-organ injury or death included maternal age, low income, world region of origin, high BMI, pre-existing medical conditions, and placental disorders (Table S3), which contributed to the final model. Attendance at a first-trimester antenatal visit and parity were inversely associated with the composite outcome. The corresponding model C-statistic was 0.68 (95% CI 0.66–0.69) (Fig. 1). There was minimal overfitting of the model, with mean optimism of 0.0055 (95% CI 0.0050–0.0061), and an optimism-corrected C-statistic of 0.67 (95% CI 0.66–0.68). Model discrimination was unchanged when BMI was included, either as a categorical variable with “missing” as a separate category, or as a continuous variable imputed with the median value for BMI. We tested 300 pairwise interactions, of which 13 interactions were statistically significant. The main model including interaction terms resulted in similar model discrimination as the main model (C-statistic 0.69, 95% CI 0.68–0.70), however this model included unstable estimates. Therefore, the model without interaction terms was chosen as the most balanced and efficient model.

All-cause mortality from birth until 365 days postpartum occurred in 194 women over the study time period (0.3 per 1000). The final multivariable model for all-cause mortality no longer retained world region of origin, parity, previous spontaneous abortion, and several medical comorbidities (Table S4). Major psychiatric conditions and alcohol and substance use newly emerged as predictors. The corresponding C-statistic was 0.70 (95% CI 0.66–0.74) (Figure S2). However, this model was slightly over-fitted, and the optimism-corrected C-statistic was 0.67 (95% CI 0.63–0.71).

The risk classification table for the main model, dividing the cohort according to the five categories of predicted risk of acute end-organ injury or death (Table 3) demonstrated the capacity of this model to classify women who are at very low risk (−LR 0.41, 95% CI 0.33–0.52) and those at very high risk of the outcome (+LR 8.58, 95% CI 7.32–10.05), but was less useful in classifying women in intermediate risk categories.

**Table 3:** Risk classification comparing predicted and observed risks of the outcome using five groups of predicted probability, and associated likelihood ratios in each group. Data are from main model predicting acute end organ injury or death from 20 weeks' gestation until 42 days after birth (*n* = 634,290)

**Predicted risk group** (per 1000)	**Observed acute end-organ injury or death**			
	**Yes** **n (%)**	**No** **n (%)**	**Likelihood ratio**	**95% CI**
**Very low risk** < 1.5	72 (3.66)	55,957 (8.85)	0.41	0.33–0.52
**Low risk** 1.5 to 3.0	834 (42.36)	403,060 (63.74)	0.66	0.61–0.72
**Intermediate risk** 3.0 to 5.0	408 (20.72)	108,085 (17.09)	1.21	1.09–1.35
**High risk** 5.0 to 15.0	485 (24.63)	58,854 (9.31)	2.65	2.40–2.92
**Very high risk** > 15.0	170 (8.63)	6365 (1.01)	8.58	7.32–10.05
**Total**	1969 (100)	632,321 (100)		

#### Sub-cohort of parous women

**Fig. 2:**
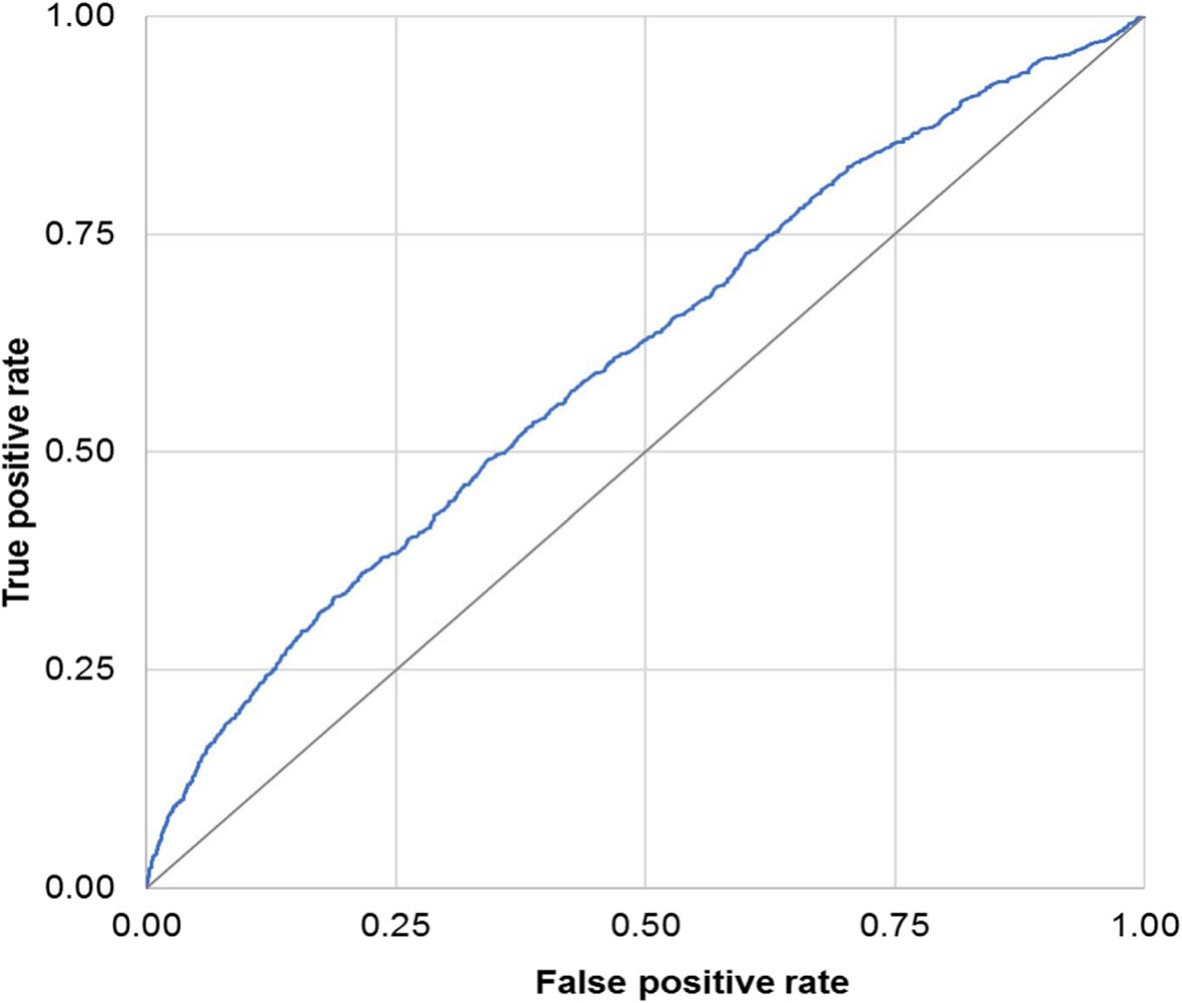
Receiver operating characteristic curve showing the discrimination of the clinical prediction model for maternal end-organ injury or death. Legend: Outcomes are those arising between 20 weeks’ gestation and 42 days after birth using variables measured in the index pregnancy prior to 20 weeks’ gestation (**a**); the index pregnancy prior to 20 weeks’ gestation and pre-pregnancy (**b**); the index pregnancy prior to 20 weeks’ gestation, pre-pregnancy, and in a previous pregnancy (**c**). Predictor variables and odds ratios are shown in Tables S5 (**a**), S6 (**b**), and S7 (**c**). Analysed is the cohort of 333,435 births among parous women. **a** C-statistic for Area Under the Curve = 0.61 (95% CI 0.59–0.63). **b** C-statistic for Area Under the Curve = 0.69 (95% CI 0.67–0.70). **c** C-statistic for Area Under the Curve = 0.71 (95% CI 0.69–0.73)

In the sub-cohort of 333,435 parous women, the aforementioned variables significantly associated with end-organ injury or death persisted, as did the addition of an unplanned Caesarean delivery and severe organ injury in a previous birth (Table S7). The C-statistic was 0.61 (95% CI 0.59–0.63) when limited to variables from the index pregnancy (Fig. 2a), rising to 0.69 (95% CI 0.67–0.70) after adding pre-pregnancy predictors (Fig. 2b), and 0.71 (95% CI 0.69–0.73) when including the variables from a previous pregnancy (Fig. 2c). We noted minimal overfitting for each model. With optimism-corrected C-statistics of 0.60 (95% CI 0.58–0.62), 0.68 (95% CI 0.66–0.70), and 0.70 (95% CI 0.69–0.72), respectively.

### Model fit and calibration

Visual inspection of the calibration plots in the entire cohort suggested good agreement between observed and expected events for the primary outcome, with slightly worse calibration for mortality (Figure S3a-b). Among parous women, model calibration for maternal end-organ injury or death improved from the base model to models including variables measured pre-pregnancy and in a previous pregnancy (Figure S3c-e).

## Discussion

### Main findings

We have shown that a model based on variables available pre-pregnancy and in early pregnancy can moderately discriminate women destined for a severely morbid event or death from those likely to have uncomplicated pregnancies. Predictive variables retained in the final models included demographic, obstetric, and other medical risk factors. Notably, attendance at the first trimester visit with a care provider – a measure of good prenatal care – was inversely associated with the risk of SMM. Inclusion of prior pregnancy factors, which have not been incorporated in previous predictive models for SMM, further enhanced model performance, in keeping with the importance of clinical obstetrical history. Our model displayed good calibration, indicating that a combination of routinely measured pre-pregnancy and early pregnancy factors can estimate the absolute risk of acute end-organ injury or death with reasonable accuracy. Using this model effectively increased the probability of identifying a very high-risk woman with this outcome by 40%, and reduced the probability in someone considered very low-risk by 20% [33], but was less useful in classifying women in intermediate risk categories. This suggests that additional clinical, laboratory, or paraclinical factors are needed to accurately predict morbidity in all women, and further, that a certain proportion of these events are truly sudden and unpredictable.

### Strengths and limitations

The models in this study relied on information that is routinely known at the time of the first antenatal visit, using variables that were temporally remote from when most maternal morbid events arise – largely around the time of birth [1]. Moreover, our source population comprised all pregnancies from gestational week 20. However, our datasets had few routinely collected clinical measures, such as blood pressure and haemoglobin or glucose concentrations, or first-trimester screening biomarkers. In the prediction of preterm preeclampsia, for example, a model that contained a combination of clinical and paraclinical variables (including placental biomarkers) performed better than with either set of variables in isolation [34]. BMI was incomplete in our dataset, as is common in most administrative data sources. However, the proportion in any given BMI category and those with missing values was not appreciably different among women with and without the outcome. Furthermore, there were few substantial differences in other baseline characteristics between those with missing vs. non-missing BMI (Table S8). Thus, while the contribution of BMI to the outcome may not have been well represented in our models, this unlikely changed the overall model performance. In addition, clinical practice around identification and management of SMM has evolved over time. It is plausible that the strength of different risk factors for SMM may have changed across the study period as well (e.g., use of lower-risk IVF strategies). However, we used a constant definition for the study outcomes, and any changes in clinical practice patterns would not affect the internal validity of our models.

Prediction models are often used to estimate an individual’s absolute risk of a serious adverse event that might be mitigated with the use of a particular therapy, while avoiding subjecting individuals at low predicted risk to potential harmful effects of such therapy [35]. In obstetrics, serious adverse events are rare, with limited options for targeted prevention. We acknowledge, therefore, the limitations afforded by the C-statistic to discriminate between individuals with and without a rare adverse event, in which a high false positive rate might be justified [36]. The LRs add clinical meaning to the model and serve as a foundation for what might be considered reasonable predictability of rare but catastrophic obstetric events. The high LR of the model for women with very high predicted risk despite the rarity of the outcome in this group speaks to the potential utility of the model as a screening tool.

### Interpretation

The models in this study relied on information that is routinely known at the time of the first antenatal visit, and that is temporally remote from when most morbid events arise – around the time of birth [1]. Our main model shows the potential utility of harnessing data in early pregnancy to predict a variety of later adverse maternal outcomes. Consistent with previous research on postnatal mortality [23], our model for all-cause mortality showed substance use, alcohol use, and psychiatric conditions to be significant predictors of death up to 365 days postpartum.

SMM rates have stagnated within Western nations, yet evidence-based strategies to reduce their burden are lacking [1]. Despite the possibility for early identification and prevention of some forms of SMM, current practice guidelines do not incorporate recommendations for prediction of severe morbidity, and use narrow crude definitions to identify such events [37]. Further refinement of clinical prediction models and the eventual development of a clinical risk calculator may help to inform early triage of women for enhanced surveillance or referral to subspecialty care or shared-care antenatal pathways – decisions that at present rely principally on clinical judgment. In developing and refining our study’s model in external cohorts, investigators should consider adding first-trimester placental biomarkers and other maternal biomarkers alongside routinely measured clinical variables, such as blood pressure and weight. The incorporation of such variables may facilitate prediction of the whole of severe morbidity as well as cause-specific outcomes, and better inform individualized and targeted prevention [38].

## Conclusion

In conclusion, a model developed using pre-pregnancy and early pregnancy predictors available within administrative datasets had moderate prediction of maternal acute end-organ injury or death, and as such shows significant promise in the early clinical prediction of SMM. The addition of factors from a prior pregnancy among parous women slightly improved the model performance. Enhancement of these models, using direct clinical measures, and by external validation or using machine learning, is needed.

## Supplementary Information

**Additional file 1: Table S1.** Variables used to define cohort entry and exclusion criteria, primary outcome, and variables considered for each clinical prediction model. **Table S2.** List of countries used to define World region of origin among immigrant women included in the study. **Table S3.** Adjusted odds ratio of maternal end-organ injury or death arising between 20 weeks’ gestation and up to 42 days after birth, in association with variables measured pre-pregnancy and in the index pregnancy prior to 20 weeks’ gestation. **Table S4.** Adjusted odds ratio of all-cause maternal death arising between birth and up to 365 days after birth, in association with variables measured pre-pregnancy and in the index pregnancy prior to 20 weeks’ gestation. **Table S5.** Adjusted odds ratio of maternal end-organ injury or death arising between 20 weeks’ gestation and up to 42 days after birth in association with variables measured in the index pregnancy prior to 20 weeks’ gestation. **Table S6.** Adjusted odds ratio of maternal end-organ injury or death arising between 20 weeks’ gestation and up to 42 days after birth in association with variables measured in the index pregnancy prior to 20 weeks’ gestation and pre-pregnancy. **Table S7.** Adjusted odds ratio of maternal end-organ injury or death arising between 20 weeks’ gestation and up to 42 days after birth in association with variables measured in the index pregnancy prior to 20 weeks’ gestation, pre-pregnancy, and in any previous pregnancy. **Table S8.** Baseline characteristics of the study population, according to missing BMI vs. non-missing BMI. **Figure S1.** Flow diagram of the creation of the study cohort. **Figure S2.** Receiver operating characteristic curve showing discrimination of the clinical prediction model for all-cause maternal death. **Figure S3.** a. Calibration plot of predicted (x-axis) and observed (y-axis) deciles of probability of acute end organ injury or death, entire cohort of 634,290 births. b. Calibration plot of predicted (x-axis) and observed (y-axis) deciles of probability of all-cause maternal death, entire cohort of 634,290 births. c. Calibration plot of predicted (x-axis) and observed (y-axis) deciles of probability of acute end organ injury or death, early index pregnancy factors, sub-cohort of 333,435 births among parous women. d. Calibration plot of predicted (x-axis) and observed (y-axis) deciles of probability of acute end organ injury or death, pre-pregnancy and early index pregnancy factors, sub-cohort of 333,435 births among parous women. e. Calibration plot of predicted (x-axis) and observed (y-axis) deciles of probability of acute end organ injury or death, pre-pregnancy, previous pregnancy, and early index pregnancy factors, sub-cohort of 333,435 births among parous women.

## Data Availability

The dataset from this study is held securely in coded form at ICES. While data sharing agreements prohibit ICES from making the dataset publicly available, access may be granted to those who meet pre-specified criteria for confidential access, available at www.ices.on.ca/DAS. The full dataset creation plan and underlying analytic code are available from the authors upon request, understanding that the computer programs may rely upon coding templates or macros that are unique to ICES and are therefore either inaccessible or may require modification.

## References

[1] Ray JG, Park AL, Dzakpasu S (2018). Prevalence of SMM and factors associated with maternal mortality in Ontario, Canada. JAMA Netw Open.

[2] Lindquist A, Knight M, Kurinczuk JJ (2013). Variation in SMM according to socioeconomic position: a UK national case-control study. BMJ Open.

[3] Dayan N, Fell DB, Guo Y (2018). SMM in women with high BMI in IVF and unassisted singleton pregnancies. Hum Reprod.

[4] D'Alton ME, Bonanno CA, Berkowitz RL (2013). Putting the “M” back in maternal-fetal medicine. Am J Obstet Gynecol.

[5] van Roosmalen J, Zwart J (2009). Severe acute maternal morbidity in high-income countries. Best Pract Res Clin Obstet Gynaecol.

[6] Firoz T, Chou D, von Dadelszen P (2013). Measuring maternal health: focus on maternal morbidity. Bull World Health Organ.

[7] Malhame I, Danilack VA, Raker CA, et al. Cardiovascular SMM in pregnant and postpartum women: development and internal validation of risk prediction models. BJOG. 2021;128(5):922–32. 10.1111/1471-0528.16512 32946639

[8] Aoyama K, Pinto R, Ray JG (2019). Association of maternal age with SMM and mortality in Canada. JAMA Netw Open.

[9] Bateman BT, Mhyre JM, Hernandez-Diaz S (2013). Development of a comorbidity index for use in obstetric patients. Obstet Gynecol.

[10] Metcalfe A, Lix LM, Johnson J-A, Currie G, Lyon AW, Bernier F (2015). Validation of an obstetric comorbidity index in an external population. BJOG.

[11] Aoyama K, D'Souza R, Pinto R (2018). Risk prediction models for maternal mortality: a systematic review and meta-analysis. PLoS One.

[12] Menard MK (2019). Toward achieving risk-appropriate maternity care: maternal morbidity prediction. Obstet Gynecol.

[13] Macones GA (2013). Understanding and reducing serious maternal morbidity: a step in the right direction. Obstet Gynecol.

[14] Dayan N, Joseph KS, Fell DB (2019). Infertility treatment and risk of SMM: a propensity score-matched cohort study. CMAJ.

[15] Collins GS, Reitsma JB, Altman DG, Moons KG (2015). Transparent reporting of a multivariable prediction model for individual prognosis or diagnosis (TRIPOD): the TRIPOD statement. BMJ.

[16] BORN Ontario (2019). Legacy data elements.

[17] Dunn S, Lanes A, Sprague AE (2019). Data accuracy in the Ontario birth registry: a chart re-abstraction study. BMC Health Serv Res.

[18] Dunn S, Bottomley J, Ali A, Walker M (2011). 2008 Niday Perinatal Database quality audit: report of a quality assurance project. Chronic Dis Inj Can.

[19] Joseph KS, Liu S, Rouleau J, Kirby RS, Kramer MS, Sauve R (2010). SMM in Canada, 2003 to 2007: surveillance using routine hospitalization data and ICD-10CA codes. J Obstet Gynaecol Can.

[20] Main EK, Abreo A, McNulty J (2016). Measuring SMM: validation of potential measures. Am J Obstet Gynecol.

[21] Roberts CL, Cameron CA, Bell JC, Algert CS, Morris JM (2008). Measuring maternal morbidity in routinely collected health data: development and validation of a maternal morbidity outcome indicator. Med Care.

[22] Grigoriadis S, Wilton AS, Kurdyak PA (2017). Perinatal suicide in Ontario, Canada: a 15-year population-based study. CMAJ.

[23] Ray JG, Zipursky J, Park AL (2018). Injury-related maternal mortality. Am J Obstet Gynecol.

[24] Hair JF, Black WC, Babin BJ, Anderson RE, Tatham RL (2006). Multivariate data analysis.

[25] Steyerberg EW. Clinical prediction models: A practical approach to development, validation, and updating. New York: Springer; 2009.

[26] Austin PC (2009). Using the standardized difference to compare the prevalence of a binary variable between two groups in observational research. Commun Stat Simul Comput.

[27] Hanley JA, McNeil BJ (1982). The meaning and use of the area under a receiver operating characteristic (ROC) curve. Radiology.

[28] Steyerberg EW (2009). Mathematics and statistics. Clinical prediction models: a practical approach to development, validation, and updating.

[29] Steyerberg EW, Harrell FE, Borsboom GJ, Eijkemans MJ, Vergouwe Y, Habbema JD (2001). Internal validation of predictive models: efficiency of some procedures for logistic regression analysis. J Clin Epidemiol.

[30] Harrell FE, Lee KL, Mark DB (1996). Multivariable prognostic models: issues in developing models, evaluating assumptions and adequacy, and measuring and reducing errors. Stat Med.

[31] Steyerberg EW, Vickerrs AJ, Cook NR, Gerds T, Gonen M, Obuchowski N (2010). Assessing the performance of prediction models: a framework for some traditional and novel measures. Epidemiology.

[32] Deeks JJ, Altman DG (2004). Diagnostic tests 4: likelihood ratios. Br Med J.

[33] McGee S (2002). Simplfying likelihood ratios. J Gen Intern Med.

[34] Akolekar R, Syngelaki A, Poon L, Wright D, Nicolaides KH (2013). Competing risks model in early screening for preeclampsia by biophysical and biochemical markers. Fetal Diagn Ther.

[35] Alba AC, Agoritsas T, Walsh M (2017). Discrimination and calibration of clinical prediction models: users’ guides to the medical literature. JAMA.

[36] Romero-Brufau S, Huddleston JM, Escobar GJ, Liebow M (2015). Why the C-statistic is not informative to evaluate early warning scores and what metrics to use. Crit Care (London, England).

[37] Kilpatrick SK, Ecker JL, American College of O, Gynecologists, the Society for Maternal-Fetal M (2016). SMM: screening and review. Am J Obstet Gynecol.

[38] Davidson AJ, Park AL, Ray JG (2019). Navigating SMM using big data: green, yellow, and red flags for researchers. Obstet Med.

