# Supplementary material for: Development and internal validation of a model predicting severe maternal morbidity using pre-conception and early pregnancy variables: a population-based study in Ontario, Canada
Source: BMC Pregnancy Childbirth. 2021 Oct 6;21:679. doi: 10.1186/s12884-021-04132-6 (PMC8496026; doi:10.1186/s12884-021-04132-6)
Supplement: Supplementary file 1 — Additional file 1: Table S1. Variables used to define cohort entry and exclusion criteria, primary outcome, and variables considered for each clinical prediction model. Table S2. List of countries used to define World region of origin among immigrant women included in the study. Table S3. Adjusted odds ratio of maternal end-organ injury or death arising between 20 weeks’ gestation and up to 42 days after birth, in association with variables measured pre-pregnancy and in the index pregnancy prior to 20 weeks’ gestation. Table S4. Adjusted odds ratio of all-cause maternal death arising between birth and up to 365 days after birth, in association with variables measured pre-pregnancy and in the index pregnancy prior to 20 weeks’ gestation. Table S5. Adjusted odds ratio of maternal end-organ injury or death arising between 20 weeks’ gestation and up to 42 days after birth in association with variables measured in the index pregnancy prior to 20 weeks’ gestation. Table S6. Adjusted odds ratio of maternal end-organ injury or death arising between 20 weeks’ gestation and up to 42 days after birth in association with variables measured in the index pregnancy prior to 20 weeks’ gestation and pre-pregnancy. Table S7. Adjusted odds ratio of maternal end-organ injury or death arising between 20 weeks’ gestation and up to 42 days after birth in association with variables measured in the index pregnancy prior to 20 weeks’ gestation, pre-pregnancy, and in any previous pregnancy. Table S8. Baseline characteristics of the study population, according to missing BMI vs. non-missing BMI. Figure S1. Flow diagram of the creation of the study cohort. Figure S2. Receiver operating characteristic curve showing discrimination of the clinical prediction model for all-cause maternal death. Figure S3. a. Calibration plot of predicted (x-axis) and observed (y-axis) deciles of probability of acute end organ injury or death, entire cohort of 634,290 births. b. Calibration plot of predicted (x-axis [file 12884_2021_4132_MOESM1_ESM.docx]

**Title: Development and internal validation of a model predicting severe maternal morbidity using pre-conception and early pregnancy variables: a population-based study in Ontario, Canada**

**Supplementary Material**

Natalie DAYAN MD, Gabriel D SHAPIRO PhD, Jin LUO MSc, Jun GUAN MSc, Deshayne B FELL PhD, Carl A LASKIN MD, Olga BASSO PhD, Alison L PARK MSc, Joel G RAY MD

**Table of contents**

Table S1. Variables used to define cohort entry and exclusion criteria, primary outcome, and variables considered for each clinical prediction model.

Table S2. List of countries used to define World region of origin among immigrant women included in the study.

Table S3. Adjusted odds ratio of maternal end-organ injury or death arising between 20 weeks’ gestation and up to 42 days after birth, in association with variables measured pre-pregnancy and in the index pregnancy prior to 20 weeks’ gestation.

Table S4. Adjusted odds ratio of all-cause maternal death arising between birth and up to 365 days after birth, in association with variables measured pre-pregnancy and in the index pregnancy prior to 20 weeks’ gestation.

Table S5. Adjusted odds ratio of maternal end-organ injury or death arising between 20 weeks’ gestation and up to 42 days after birth in association with variables measured in the index pregnancy prior to 20 weeks’ gestation.

Table S6. Adjusted odds ratio of maternal end-organ injury or death arising between 20 weeks’ gestation and up to 42 days after birth in association with variables measured in the index pregnancy prior to 20 weeks’ gestation and pre-pregnancy.

Table S7. Adjusted odds ratio of maternal end-organ injury or death arising between 20 weeks’ gestation and up to 42 days after birth in association with variables measured in the index pregnancy prior to 20 weeks’ gestation, pre-pregnancy, and in any previous pregnancy.

Table S8. Baseline characteristics of the study population, according to missing BMI vs. non-missing BMI.

Figure S1. Flow diagram of the creation of the study cohort.

Figure S2. Receiver operating characteristic curve showing discrimination of the clinical prediction model for all-cause maternal death.

Figure S3a. Calibration plot of predicted (x-axis) and observed (y-axis) deciles of probability of acute end organ injury or death, entire cohort of 634,290 births.

Figure S3b. Calibration plot of predicted (x-axis) and observed (y-axis) deciles of probability of all-cause maternal death, entire cohort of 634,290 births.

Figure S3c. Calibration plot of predicted (x-axis) and observed (y-axis) deciles of probability of acute end organ injury or death, early index pregnancy factors, sub-cohort of 333,435 births among parous women.

Figure S3d. Calibration plot of predicted (x-axis) and observed (y-axis) deciles of probability of acute end organ injury or death, pre-pregnancy and early index pregnancy factors, sub-cohort of 333,435 births among parous women.

Figure S3e. Calibration plot of predicted (x-axis) and observed (y-axis) deciles of probability of acute end organ injury or death, pre-pregnancy, previous pregnancy, and early index pregnancy factors, sub-cohort of 333,435 births among parous women.

**Table S1. Variables used to define cohort entry and exclusion criteria, primary outcome, and variables considered for each clinical prediction model.**

| **Assessment** | **Timing** | **Disease or procedure or condition** | **ICD-10-CA codes and Canadian Classification of Intervention (CCI) codes or CIHI-DAD variables** | **OHIP ICD-9 diagnostic codes or fee codes [or other source if in brackets]** |
| --- | --- | --- | --- | --- |
| *Cohort entry criterion* | April 1, 2006 to March 31, 2014 | Women aged 18 to 60 years with a livebirth or a stillbirth delivery at ≥ 20 weeks’ gestation.  Random selection of one birth per woman from within the cohort of all livebirths and stillbirths, to create the main cohort | -- | [Better Outcome Registry and Network (BORN)] [www.bornontario.ca](http://www.bornontario.ca) |
| *Exclusion criteria* | At the time of the index delivery hospitalization | Invalid maternal record | -- | [BORN] |
|  | Same | Invalid OHIP number | -- | [Registered Persons Database (RPDB) contains demographic information and encrypted healthcare numbers for all individuals eligible for OHIP] |
|  | Same | Maternal age < 18 or > 60 years, or missing | -- | [RPDB] |
|  | Same | Non-Ontario resident | -- | [RPDB] |
|  | Same | Gestational age < 20 weeks or missing | -- | [BORN] |
|  | Same | Ectopic pregnancy | O00.x | -- |
|  | Same | Pregnancy terminated by induced or therapeutic abortion | M_NONSP_STILLBIRTH = ‘T’ or M_TOP = ‘T’ in MOMBABYSB2015 (see <https://datadictionary.ices.on.ca/Applications/DataDictionary/Library.aspx?Library=MOMBABY>) | -- |
| *Primary outcome: Maternal end-organ injury or death* | From 20 weeks’ gestation until 42 days after the birth hospital discharge date | Acute heart failure | I26.0, I46, I50, I97.8, I97.9, O75.4 |  |
|  | Same | Acute liver disease | K71, K72, O26.6 |  |
|  | Same | Acute myocardial infarction | I21, I22 |  |
|  | Same | Acute renal failure | N17, N19, N99.0, O90.4 |  |
|  | Same | Adult respiratory distress syndrome/respiratory failure | J80 |  |
|  | Same | Cerebral edema or coma | G93.6, R40.2 |  |
|  | Same | Acute psychosis/delirium | F23, F53.1 |  |
|  | Same | Disseminated intravascular coagulation | D65 |  |
|  | Same | Puerperal cerebrovascular disorders | G08, G43, G93.1, G93.4, G97.8, G97.9, I60-I63, I67.4, I67.6, I67.8, I97.8, I97.9, O22.5, O22.8, O22.9, O87.3, O99.4 |  |
|  | Same | Left ventricular failure | I50.1 |  |
|  | Same | Sepsis | A40, A41, B37.7, O75.3, R57.2, R65.1 |  |
|  | Same | Obstetric shock | O75.1, R57, T80.5, T88.6 |  |
|  | Same | Status asthmaticus | J45.01, J45.11, J45.81, J45.91 |  |
|  | Same | Status epilepticus | G41 |  |
|  | Same | Death, obstetric, cause unspecified | O95 |  |
|  | Same | Death from sequelae of direct obstetric causes | O97 |  |
|  | Same | Death, obstetric, after 42 days but 1 year after delivery | O96 |  |
|  | Same | Sudden death, death from unspecified cause | R96, R97, R98, or R99 |  |
|  | Same | Assisted ventilation through endotracheal tube | 1.GZ.31.CA-ND |  |
|  | Same | Assisted ventilation through tracheostomy | 1.GZ.31.CR-ND |  |
|  | Same | Dialysis | 1.PZ.21 |  |
| *Variables considered in the derivation of the clinical prediction models* | In the index birth, prior to 19 weeks’ gestation | Age at date of conception (years) | -- | [RPDB] |
|  | Same | Residential Income Quintile | -- | [Statistics Canada census data] |
|  | Same | World region of origin: Western Nations and Europe, Hispanic America, Caribbean, Sub-Saharan Africa, Middle East and North Africa, East Asia and Pacific, South Asia.  (See Table S2 for complete definition) | -- | [Immigration, Refugees and Citizenship Canada (IRCC)’s Permanent Resident Database] |
|  | Same | First-trimester visit |  | [BORN] |
|  | Same | Infertility treatment, including *in vitro* fertilization (IVF), intracytoplasmic sperm injection (ICSI), intrauterine insemination (IUI), ovulation induction | -- | [BORN] |
|  | Same | Parity | PREVBIRTH in CIHI-DAD | [BORN] |
|  | Same | Gestational age at delivery | -- | [BORN] |
|  | Same | Body mass index | -- | [BORN] |
|  | Same | Infertility: |  |  |
|  |  | Infertility diagnosis | N97 | 628 |
|  |  | Endometriosis | N80.0-N80.4 | 615, 617 |
|  |  | Polycystic ovarian syndrome | E28.2 | -- |
|  | Same | Infertility treatment complication: |  |  |
|  |  | Severe ovarian hyperstimulation syndrome | N98.1 | -- |
|  |  | Complications due to infertility treatment | N98.0, N98.2, N98.3, N98.8, N98.9 | -- |
|  | Same | Multiple gestation | O30.0-O30.2, O30.8-O30.9, O31 | 651 or [BORN] |
|  | Same | Placental disorder: |  |  |
|  |  | Placenta praevia | O44.0-O44.1 | 641 or [BORN] |
|  |  | Placenta accrete | O43.201, O43.203, O43.209 | -- |
|  |  | Vasa previa | O69.4 | -- |
|  |  | Other placental disorders | O43.1, O43.2, O43.80, O43.81, O43.88, O43.9 | -- |
| *Complications in any previous livebirth or stillbirth delivery* | Hospitalization for delivery in any prior birth | Unplanned caesarean delivery | 5.MD.60.^^ | [BORN] |
|  | Same | Severe organ injury | All non-fatal components of primary outcome |  |
|  | Same | Preeclampsia, eclampsia, HELLP | O11, O14, O15 | [BORN] |
|  | Same | Preterm delivery < 37 weeks’ gestation | B_GESTWKS_DEL < 37, else M_GESTWKS_DEL < 37, else M_GESTWKS_ADM < 37 | [BORN] |
|  | Same | Gestational diabetes | ICD-10-CA codes E10, E11, E13, E14 **- O24**, OR 2 or more OHIP physician fee-for-service billing claims bearing a diagnosis of diabetes in the last 120 days of pregnancy (i.e., from 23 wk. gestation onward) | [BORN] |
|  | Same | Previous spontaneous abortion | O03.0-O03.9, or PREVSPABORT | -- |
| *Pre-existing medical conditions* | Within 365 days before the estimated date of conception in the index birth | Obesity (body mass index > 30 kg/m^2^) | E66 | 278 or [BORN] |
|  | Same | Systemic lupus erythematosus | M32, M32.0, M32.1 (I39.-, I32.8, N08.5, N16.4, J99.1), M32.8 | [BORN] |
|  | Same | Chronic congestive heart failure^(a)^ | I50 | 428 or [BORN] |
|  | Same | Congenital heart disease^(a)^ | Q20-Q26 | 745-747 |
|  | Same | Pulmonary hypertension^(a)^ | I27.0, I27.2 | -- |
|  | Same | Coronary artery disease^(a)^ | I20, I21, I24, I25.0, I25.1, I51.3, 1HZ80, 1IJ50, 1IJ55, 1IJ57, 1IJ76, 1IJ80, 1IK80, 1IK87, 1IL35, 2IL70, 3IP10 | 410, 412, 413, 429 |
|  | Same | Cardiac dysrhythmia^(a)^ | I48, I47.2, I49.0, | 427 |
|  | Same | Chronic rheumatic heart disease^(a)^ | I05-I09 | 398 |
|  | Same | Previous stroke^(a)^ | G46, I63.0-I66.9, I67.2, I67.8 | 432, 436, 437 |
|  | Same | Chronic hypertension | I10, I11-I15 | 401 or [BORN] |
|  | Same | Dyslipidemia | E78 | 272 |
|  | Same | Renal disease | N17.x, O08.4, T79.5, O90.4, E10.20, E10.21, E10.23, E11.20, E11.21, E11.23, M10.39, I12, I13, I15.0, I70.1, M31.0, N01.x, N03.x, N04.x, .N05.x, N06.x, N07.x, N08.x, N11.x, N12, N13.7, N13.8, N13.9, N14.x, N15.x, N16.x, N18.x, N19.x, N25.0, N25.8, N25.9, N26, R80, R94 | 403, 58 |
|  | Same | Diabetes mellitus | E10-E14 | 250 or [BORN] |
|  | Same | Liver disease | K70-K77 | 571, 573 |
|  | Same | Major psychiatric condition | F20, F30, F31, F32, F33, F34, F40, F41 | 311, 300, 295, 296, 297 or [BORN] |
|  | Same | Alcohol abuse | Z71.4, Z86.40, F10.0, F10.1, F10.2, F10.3, F10.4, F10.5, F10.6, F10.7, F10.8, F10.9 | 291, 303 or [BORN] |
|  | Same | Drug abuse | Z71.5, Z86.41, Z72.2 | 292, 304 or [BORN] |
|  | Same | Tobacco use | -- | 305 or [BORN] |
|  | Same | Asthma | J45-J46 | 493 or [BORN] |
|  |  | Sickle cell disease^(b)^ | D57.1, D57.2, D57.3, D57.8 | -- |
|  |  | HIV^(b)^ | B24, O98.701 (Z21, R75) | 042, 043, 044 or [BORN] |

^(a)^Combined under a “cardiovascular morbidity composite measure”

^(b)^Combined under “other medical conditions”

**Table S2. List of countries used to define World region of origin among immigrant women included in the study.**

| **Western Nations and Europe**: Albania, Andorra, Australia, Austria, Belarus, Belgium, Bermuda, Bosnia and Herzegovina, Bulgaria, Croatia, Czech Republic, Denmark, Estonia, Finland, Former Czechoslovakia, Former USSR, Former Yugoslavia, Falkland Islands, France, Germany, Greece, Greenland, Hungary, Iceland, Ireland, Italy, Kosovo, Latvia, Liechtenstein, Lithuania, Luxembourg, Macedonia, Malta, Moldova, Monaco, Montenegro, Netherlands, New Zealand, Norway, Pitcairn Island, Poland, Portugal, Romania, Russian Federation, San Marino, Serbia, Serbia and Montenegro, Slovakia, Slovenia, Southern Antarctic Territories, Spain, St. Helena, St. Pierre and Miquelon, Sweden, Switzerland, Ukraine, United Kingdom, United States, Vatican City State |
| --- |
| **Hispanic America**: Argentina, Bolivia, Brazil, Chile, Colombia, Costa Rica, Cuba, Dominican Republic, Ecuador, El Salvador, French Guiana, Guatemala, Guyana, Honduras, Mexico, Nicaragua, Panama, Paraguay, Peru, Puerto Rico, Suriname, Uruguay, Venezuela |
| **Caribbean**: Anguilla, Antigua and Barbuda, Aruba, Bahamas, Barbados, Belize, Cayman Islands, Dominica, Grenada, Guadeloupe, Haiti, Jamaica, Martinique, Montserrat, Netherlands Antilles, Saint Kitts and Nevis, Saint Lucia, Saint Vincent And The Grenadines, Trinidad and Tobago, Turks and Caicos Islands, Virgin Islands, British Virgin Islands, West Indies NES |
| **Sub-Saharan Africa**: Angola, Benin, Botswana, Burkina Faso, Burundi, Cameroon, Cape Verde, Central African Republic, Chad, Comoros, Congo, The Democratic Republic of Congo, Cote D'ivoire, Djibouti, Equatorial Guinea, Eritrea, Ethiopia, Gabon, Gambia, Ghana, Guinea, Guinea Bissau, Kenya, Lesotho, Liberia, Madagascar, Malawi, Mali, Mauritania, Mauritius, Mayotte, Mozambique, Namibia, Niger, Nigeria, Reunion, Rwanda, Sao Tome and Principe, Senegal, Seychelles, Sierra Leone, Somalia, South Africa, Swaziland, Tanzania, United Republic Of Togo, Uganda, Western Sahara, Zambia, Zimbabwe |
| **Middle East and North Africa**: Algeria, Armenia, Azerbaijan, Bahrain, Cyprus, Egypt, Georgia, Iran, Iraq, Israel, Jordan, Kazakhstan, Kuwait, Kyrgyzstan, Lebanon, Libya, Morocco, Oman, Occupied Palestinian Territory, Qatar, Saudi Arabia, Sudan, Syria, Tajikistan, Tunisia, Turkey, Turkmenistan, United Arab Emirates, Uzbekistan, Yemen |
| **East Asia and Pacific**: Asia NES, Brunei Darussalam, Cambodia, China, Fiji, French Polynesia, Guam, Hong Kong, Indonesia, Japan, Kiribati, Korea North, Korea South, Laos, Macau, Malaysia, Mongolia, Myanmar, Nauru, New Caledonia, Northern Mariana Islands, Palau, Papua New Guinea, Philippines, Samoa, Singapore, Solomon Islands, Taiwan, Thailand, Tibet, Tonga, Vanuatu, Viet Nam, Tuvalu, Marinas, Republic of The Marshall Islands, Federated States of Micronesia, Cook Islands, Wallis And Futuna, Ocean NES, East Timor |
| **South Asia**: Afghanistan, Bangladesh, Bhutan, India, Maldives, Nepal, Pakistan, Sikkim, Sri Lanka |

**Table S3. Adjusted odds ratio of maternal end-organ injury or death arising between 20 weeks’ gestation and up to 42 days after birth, in association with variables measured pre-pregnancy and in the index pregnancy prior to 20 weeks’ gestation.** Analysed is the entire cohort of 634,290 births.

| **Variable** | **Adjusted odds ratio (95% CI)** |
| --- | --- |
| Age at conception, 17-24 vs. 25-29 years | 0.88 (0.76 to 1.03) |
| Age at conception, 30-34 vs. 25-29 years | 1.24 (1.10 to 1.40) |
| Age at conception, 35-39 vs. 25-29 years | 1.58 (1.38 to 1.80) |
| Age at conception, ≥ 40 vs. 25-29 years | 2.03 (1.66 to 2.49) |
| Income quintile 1 (lowest) vs. 5 (highest) | 1.32 (1.13 to 1.54) |
| Income quintile 2 vs. 5 (highest) | 1.14 (0.98 to 1.34) |
| Income quintile 3 vs. 5 (highest) | 1.25 (1.07 to 1.46) |
| Income quintile 4 vs. 5 (highest) | 1.09 (0.93 to 1.28) |
| Caribbean vs. Canada^(a)^ | 1.73 (1.33 to 2.25) |
| East Asia and Pacific vs. Canada^(a)^ | 1.07 (0.89 to 1.28) |
| Hispanic America vs. Canada^(a)^ | 1.09 (0.81 to 1.46) |
| Middle East and North Africa vs. Canada^(a)^ | 0.79 (0.58 to 1.07) |
| South Asia vs. Canada^(a)^ | 1.05 (0.89 to 1.23) |
| Sub-Saharan Africa vs. Canada^(a)^ | 1.98 (1.57 to 2.49) |
| Western Nations and Europe vs. Canada^(a)^ | 0.84 (0.66 to 1.07) |
| First-trimester visit | 0.78 (0.71 to 0.86) |
| Body mass index, per kg/m^2^ | 1.03 (1.02 to 1.04) |
| Cardiovascular morbidity | 2.61 (2.17 to 3.16) |
| Systemic lupus erythematosus | 3.39 (1.95 to 5.88) |
| Chronic hypertension | 2.16 (1.82 to 2.57) |
| Renal disease | 7.10 (4.74 to 10.63) |
| Diabetes mellitus | 2.00 (1.61 to 2.48) |
| Liver disease | 2.10 (1.29 to 3.43) |
| Substance use | 1.99 (1.57 to 2.52) |
| Asthma | 1.54 (1.33 to 1.77) |
| Multiple gestation | 3.03 (2.60 to 3.55) |
| Placental disorders | 3.96 (3.36 to 4.67) |
| Previous spontaneous abortion | 1.15 (1.04 to 1.27) |
| Parity 1-2 vs. 0 | 0.74 (0.67 to 0.81) |
| Parity ≥ 3 vs. 0 | 0.81 (0.67 to 0.98) |

^(a)^ Includes long-term residents

**Table S4. Adjusted odds ratio of all-cause maternal death arising between birth and up to 365 days after birth, in association with variables measured pre-pregnancy and in the index pregnancy prior to 20 weeks’ gestation.** Analysed is the entire cohort of 634,290 births.

| **Variable** | **Adjusted odds ratio (95% CI)** |
| --- | --- |
| Age at conception, 17-24 vs. 25-34 years | 1.24 (0.87 to 1.77) |
| Age at conception, ≥ 35 vs. 25-34 years | 1.43 (1.00 to 2.04) |
| Income quintile 1 (lowest) vs. 5 (highest) | 1.55 (0.99 to 2.44) |
| Income quintile 2 vs. 5 (highest) | 1.06 (0.64 to 1.74) |
| Income quintile 3 vs. 5 (highest) | 1.04 (0.63 to 1.72) |
| Income quintile 4 vs. 5 (highest) | 0.68 (0.39 to 1.18) |
| First-trimester visit | 0.60 (0.45 to 0.80) |
| Cardiovascular morbidity | 2.48 (1.34 to 4.59) |
| Systemic lupus erythematosus | 5.77 (1.39 to 23.96) |
| Renal disease | 5.61 (1.71 to 18.38) |
| Major psychiatric condition | 1.39 (1.01 to 1.91) |
| Alcohol overuse | 4.19 (2.06 to 8.54) |
| Substance use | 4.62 (2.87 to 7.44) |
| Multiple gestation | 2.23 (1.24 to 4.01) |

**Table S5. Adjusted odds ratio of maternal end-organ injury or death arising between 20 weeks’ gestation and up to 42 days after birth in association with variables measured in the index pregnancy prior to 20 weeks’ gestation.** Analysed is the cohort of 333,435 births among parous women.

| **Variable** | **Adjusted odds ratio (95% CI)** |
| --- | --- |
| Age at conception, per year | 1.06 (1.05 to 1.08) |
| Income quintile 1 (lowest) vs. 5 (highest) | 1.55 (1.24 to 1.93) |
| Income quintile 2 vs. 5 (highest) | 1.24 (0.99 to 1.57) |
| Income quintile 3 vs. 5 (highest) | 1.39 (1.11 to 1.74) |
| Income quintile 4 vs. 5 (highest) | 1.19 (0.95 to 1.50) |
| Caribbean vs. Canada^(a)^ | 1.84 (1.32 to 2.57) |
| East Asia and Pacific vs. Canada^(a)^ | 0.92 (0.71 to 1.19) |
| Hispanic America vs. Canada^(a)^ | 0.86 (0.55 to 1.32) |
| Middle East and North Africa vs. Canada^(a)^ | 0.74 (0.50 to 1.10) |
| South Asia vs. Canada^(a)^ | 0.80 (0.63 to 1.01) |
| Sub-Saharan Africa vs. Canada^(a)^ | 1.62 (1.20 to 2.21) |
| Western Nations and Europe vs. Canada^(a)^ | 0.79 (0.56 to 1.12) |
| First-trimester visit | 0.80 (0.70 to 0.91) |
| Body mass index, per kg/m^2^ | 1.04 (1.03 to 1.06) |

^(a)^ Includes long-term residents

**Table S6. Adjusted odds ratio of maternal end-organ injury or death arising between 20 weeks’ gestation and up to 42 days after birth in association with variables measured in the index pregnancy prior to 20 weeks’ gestation and pre-pregnancy.** Analysed is the cohort of 333,435 births among parous women.

| **Variable** | **Adjusted odds ratio (95% CI)** |
| --- | --- |
| Age at conception, per year | 1.06 (1.04 to 1.07) |
| Income quintile 1 (lowest) vs. 5 (highest) | 1.40 (1.12 to 1.75) |
| Income quintile 2 vs. 5 (highest) | 1.17 (0.93 to 1.48) |
| Income quintile 3 vs. 5 (highest) | 1.35 (1.08 to 1.69) |
| Income quintile 4 vs. 5 (highest) | 1.18 (0.94 to 1.48) |
| Caribbean vs. Canada^(a)^ | 1.88 (1.34 to 2.62) |
| East Asia and Pacific vs. Canada^(a)^ | 1.04 (0.80 to 1.36) |
| Hispanic America vs. Canada^(a)^ | 0.95 (0.61 to 1.47) |
| Middle East and North Africa vs. Canada^(a)^ | 0.82 (0.55 to 1.21) |
| South Asia vs. Canada^(a)^ | 0.90 (0.71 to 1.15) |
| Sub-Saharan Africa vs. Canada^(a)^ | 1.69 (1.24 to 2.31) |
| Western Nations and Europe vs. Canada^(a)^ | 0.87 (0.61 to 1.23) |
| First-trimester visit | 0.83 (0.72 to 0.95) |
| Body mass index, per kg/m^2^ | 1.03 (1.02 to 1.05) |
| Cardiovascular morbidity^(b)^ | 2.35 (1.80 to 3.08) |
| Chronic hypertension | 2.37 (1.90 to 2.96) |
| Renal disease | 4.83 (2.45 to 9.54) |
| Diabetes mellitus | 1.57 (1.14 to 2.15) |
| Major psychiatric condition | 1.23 (1.06 to 1.43) |
| Substance use | 2.66 (1.95 to 3.61) |
| Asthma | 1.42 (1.15 to 1.76) |
| Other medical conditions^(c)^ | 2.91 (1.28 to 6.63) |
| Multiple gestation | 2.36 (1.81 to 3.07) |
| Placental disorders | 5.60 (4.55 to 6.90) |

^(a)^ Includes long-term residents

^(b)^ Includes chronic congestive heart failure, congenital heart disease, pulmonary hypertension, coronary artery disease, cardiac dysrhythmia, chronic rheumatic heart diseases, or non-incident stroke or myocardial infarction within the previous 365 days.

^(c)^ Includes sickle cell disease and HIV

**Table S7. Adjusted odds ratio of maternal end-organ injury or death arising between 20 weeks’ gestation and up to 42 days after birth in association with variables measured in the index pregnancy prior to 20 weeks’ gestation, pre-pregnancy, and in any previous pregnancy.** Analysed is the cohort of 333,435 births among parous women.

| **Variable** | **Adjusted odds ratio (95% CI)** |
| --- | --- |
| Age at conception, per year | 1.06 (1.04 to 1.07) |
| Income quintile 1 (lowest) vs. 5 (highest) | 1.47 (1.17 to 1.83) |
| Income quintile 2 vs. 5 (highest) | 1.20 (0.95 to 1.52) |
| Income quintile 3 vs. 5 (highest) | 1.37 (1.09 to 1.71) |
| Income quintile 4 vs. 5 (highest) | 1.18 (0.94 to 1.49) |
| Caribbean vs. Canada^(a)^ | 1.97 (1.41 to 2.75) |
| East Asia and Pacific vs. Canada^(a)^ | 1.09 (0.84 to 1.42) |
| Hispanic America vs. Canada^(a)^ | 0.99 (0.64 to 1.54) |
| Middle East and North Africa vs. Canada^(a)^ | 0.87 (0.59 to 1.30) |
| South Asia vs. Canada^(a)^ | 0.93 (0.73 to 1.18) |
| Sub-Saharan Africa vs. Canada^(a)^ | 1.70 (1.24 to 2.32) |
| Western Nations and Europe vs. Canada^(a)^ | 0.92 (0.65 to 1.30) |
| First-trimester visit | 0.83 (0.73 to 0.95) |
| Body mass index, per kg/m^2^ | 1.03 (1.01 to 1.04) |
| Cardiovascular morbidity^(b)^ | 2.18 (1.66 to 2.86) |
| Chronic hypertension | 2.23 (1.79 to 2.79) |
| Renal disease | 3.65 (1.81 to 7.36) |
| Diabetes mellitus | 1.47 (1.07 to 2.01) |
| Major psychiatric condition | 1.23 (1.06 to 1.42) |
| Substance use | 2.82 (2.08 to 3.84) |
| Asthma | 1.39 (1.13 to 1.72) |
| Other medical conditions^(c)^ | 2.94 (1.29 to 6.70) |
| Multiple gestation | 2.31 (1.78 to 3.01) |
| Placental disorders | 5.47 (4.44 to 6.74) |
| Previous unplanned caesarean delivery | 2.02 (1.76 to 2.31) |
| Severe organ injury in a previous birth | 7.71 (4.78 to 12.42) |

^(a)^ Includes long-term residents

^(b)^ Includes chronic congestive heart failure, congenital heart disease, pulmonary hypertension, coronary artery disease, cardiac dysrhythmia, chronic rheumatic heart diseases, or non-incident stroke or myocardial infarction within the previous 365 days.

^(c)^ Includes sickle cell disease and HIV

**Table S8. Baseline characteristics of the study population, according to missing BMI vs. non-missing BMI.** All data are shown as a number (%) unless otherwise stated.

| **Characteristic** | **BMI missing**  **(N=404,590)** | **BMI non-missing**  **(N=229,700)** | **Standardized difference** |
| --- | --- | --- | --- |
| **Demographic factors, at conception** |  |  |  |
| Age, y |  |  |  |
| Mean ± SD | 29.6 ± 5.5 | 29.6 ± 5.4 | 0 |
| 18-34 | 326,695 (80.7) | 187,059 (81.4) | 0.02 |
| 35-39 | 65,251 (16.1) | 35,667 (15.5) | 0.02 |
| 40-44 | 12,025 (3.0) | 6,616 (2.9) | 0.01 |
| ≥ 45 | 619 (0.2) | 358 (0.2) | 0 |
| Neighbourhood income quintile (Q) |  |  |  |
| Q1 (lowest) | 94,066 (23.2) | 52,402 (22.8) | 0.01 |
| Q2 | 82,817 (20.5) | 46,175 (20.1) | 0.01 |
| Q3 | 83,585 (20.7) | 45,888 (20.0) | 0.02 |
| Q4 | 81,299 (20.1) | 47,999 (20.9) | 0.02 |
| Q5 (highest) | 62,823 (15.5) | 37,236 (16.2) | 0.02 |
| World region of origin |  |  |  |
| Western Nations and Europe | 16,658 (4.1) | 11,256 (4.9) | 0.04 |
| Hispanic America | 9,256 (2.3) | 4,974 (2.2) | 0.01 |
| Caribbean | 7,162 (1.8) | 3,203 (1.4) | 0.03 |
| Sub-Saharan Africa | 8,150 (2.0) | 4,003 (1.7) | 0.02 |
| Middle East and North Africa | 11,497 (2.8) | 6,684 (2.9) | 0 |
| East Asia and Pacific | 27,113 (6.7) | 15,582 (6.8) | 0 |
| South Asia | 38,528 (9.5) | 17,537 (7.6) | 0.07 |
| Canada/Long-term resident | 286,226 (70.7) | 166,461 (72.5) | 0.04 |
|  |  |  |  |
| **Infertility history identified in the index pregnancy^(a)^** | 30,370 (7.5) | 19,115 (8.3) | 0.03 |
| Any infertility treatment | 10,801 (2.7) | 7,758 (3.4) | 0.04 |
|  |  |  |  |
| **Obstetrical factors in the index pregnancy** |  |  |  |
| Parity |  |  |  |
| 0 | 180,753 (44.7) | 120,102 (52.3) | 0.15 |
| 1-2 | 199,585 (49.3) | 98,541 (42.9) | 0.13 |
| ≥ 3 | 24,252 (6.0) | 11,057 (4.8) | 0.05 |
| Multiple gestation | 11,739 (2.9) | 6,589 (2.9) | 0 |
| Had a first-trimester prenatal visit | 275,972 (68.2) | 182,944 (79.6) | 0.26 |
| Placental disorder^(b)^ | 7,271 (1.8) | 4,909 (2.1) | 0.02 |
|  |  |  |  |
| **Obstetrical factors identified in any prior pregnancy^(c)^** |  |  |  |
| Unplanned Caesarean birth | 43,362 (19.4) | 22,183 (20.2) | 0.02 |
| Severe organ injury | 302 (0.1) | 201 (0.1) | 0 |
| Preeclampsia, eclampsia or HELLP syndrome | 3,119 (1.4) | 1,948 (1.8) | 0.03 |
| Preterm birth < 37 weeks’ gestation | 13,025 (5.8) | 7,023 (6.4) | 0.02 |
| Gestational diabetes mellitus | 8,161 (3.6) | 4,408 (4.0) | 0.02 |
| Previous spontaneous abortion |  |  |  |
| 0 | 312,105 (77.1) | 176,700 (76.9) | 0.01 |
| 1-2 | 83,854 (20.7) | 48,292 (21.0) | 0.01 |
| ≥ 3 | 8,533 (2.1) | 4,690 (2.0) | 0 |
| Missing | 98 (0.0) | 18 (0.0) | 0.01 |
| **Medical factors identified within 365 days before the estimated date of conception in the index birth** |  |  |  |
| Chronic hypertension | 11,218 (2.8) | 5,734 (2.5) | 0.02 |
| Renal disease | 375 (0.1) | 410 (0.2) | 0.02 |
| Diabetes mellitus (non-gestational) | 6,635 (1.6) | 3,662 (1.6) | 0 |
| Dyslipidemia | 8,412 (2.1) | 3,800 (1.7) | 0.03 |
| Cardiovascular morbidity ^(d)^ | 8,114 (2.0) | 4,477 (1.9) | 0 |
| Systemic lupus erythematosus | 401 (0.1) | 335 (0.1) | 0.01 |
| Asthma | 27,667 (6.8) | 15,922 (6.9) | 0 |
| Other medical conditions ^(e)^ | 383 (0.1) | 333 (0.1) | 0.01 |
| Alcohol overuse | 1,529 (0.4) | 1,139 (0.5) | 0.02 |
| Any substance use | 6,816 (1.7) | 5,555 (2.4) | 0.05 |
| Any tobacco use | 46,902 (11.6) | 25,716 (11.2) | 0.01 |

^(a)^ Includes diagnosis of infertility, endometriosis, and polycystic ovarian syndrome

^(b)^ Includes placenta praevia, placenta accreta, vasa praevia, and other placental disorders

^(c)^ Among 333,435 parous women

^(d)^ Includes chronic congestive heart failure, congenital heart disease, pulmonary hypertension, coronary artery disease, cardiac dysrhythmia, chronic rheumatic heart diseases, or non-incident stroke or myocardial infarction within the previous 365 days

^(e)^ Includes sickle cell disease and HIV

*Abbreviations:* SD standard deviation; HELLP haemolysis elevated liver enzymes low platelets

**Figure S1. Flow diagram of the creation of the study cohort.**

All livebirths and stillbirths,

April 2006 to March 2014

(N = 1,095,642)

Excluded:

- Invalid record/patient ID/death date (N = 42,812)
- Non-Ontario residents, GA < 20 weeks (N = 13,060)
- Ectopic pregnancy or induced abortion (N = 355)
- Unknown type of conception (N = 174,782)
- Invalid index hospitalization (N = 11,116)

N = 853,517

Randomly selected one birth per woman, resulting in the exclusion of 219,227 births

**Entire cohort**

**(N = 634,290)**

**Sub-cohort of multiparous women**

**(N = 333,435)**

**Figure S2. Receiver operating characteristic curve showing discrimination of the clinical prediction model for all-cause maternal death,** **entire cohort of 634,290 births.**

Outcomes are those arising from birth up to 365 days after birth using variables measured pre-pregnancy and in the index pregnancy prior to 20 weeks’ gestation. C-statistic for Area Under the Curve = 0.70 (95% CI 0.66-0.74).

**Figure S3a. Calibration plot of predicted (x-axis) and observed (y-axis) deciles of probability of acute end organ injury or death,** **entire cohort of 634,290 births.**

Outcomes are those arising between 20 weeks’ gestation and 42 days after birth for the clinical prediction model evaluating pre-pregnancy and early index pregnancy factors prior to 20 weeks’ gestation. Fitted slope = 1.05 (95% CI 0.94-1.15).

**Figure S3b.** **Calibration plot of predicted (x-axis) and observed (y-axis) deciles of probability of all-cause maternal death, entire cohort of 634,290 births.**

Outcome arising from birth up to 365 days after birth for the main clinical prediction model evaluating pre-pregnancy and early index pregnancy factors prior to 20 weeks’ gestation. Fitted slope = 0.94 (95% CI 0.76-1.14).

**Figure S3c. Calibration plot of predicted (x-axis) and observed (y-axis) deciles of probability of acute end organ injury or death,** **early index pregnancy factors,** **sub-cohort of 333,435 births among parous women.**

Outcomes are those arising between 20 weeks’ gestation and 42 days after birth for the clinical prediction model evaluating early index pregnancy factors prior to 20 weeks’ gestation in parous women. Fitted slope = 0.99 (95% CI 0.91-1.08).

**Figure S3d. Calibration plot of predicted (x-axis) and observed (y-axis) deciles of probability of acute end organ injury or death,** **pre-pregnancy and early index pregnancy factors, sub-cohort of 333,435 births among parous women.**

Outcomes are those arising between 20 weeks’ gestation and 42 days after birth for the clinical prediction model evaluating pre-pregnancy and early index pregnancy factors prior to 20 weeks’ gestation in parous women. Fitted slope = 0.98 (95% CI 0.77-1.20).

**Figure S3e. Calibration plot of predicted (x-axis) and observed (y-axis) deciles of probability of acute end organ injury or death,** **pre-pregnancy, previous pregnancy, and early index pregnancy factors, sub-cohort of 333,435 births among parous women.**

Outcomes are those arising between 20 weeks’ gestation and 42 days after birth for the clinical prediction model evaluating pre-pregnancy, previous pregnancy, and early index pregnancy factors prior to 20 weeks’ gestation in parous women. Fitted slope = 1.03 (95% CI 0.99-1.06).
